# Study on the willingness to enroll in the urban and rural residents basic medical insurance: from the perspectives of policy awareness and institutional trust

**DOI:** 10.3389/fpubh.2025.1535558

**Published:** 2025-06-24

**Authors:** Qunjun Yu, Ya He, Luyan Li, Danni Xu, Yalin Li, Qin Yin, Ye Lu, Mei He, Sha Ma

**Affiliations:** ^1^Department of Social Security, School of Humanities and Management, Kunming Medical University, Kunming, Yunnan, China; ^2^Medical Insurance Office, Dali Bai Autonomous Prefecture Psychiatric Hospital (The Second People's Hospital of Dali Bai Autonomous Prefecture), Dali, Yunnan, China; ^3^Department of Pharmacy, Second Affiliated Hospital of Kunming Medical University, Kunming, Yunnan, China

**Keywords:** URRBMI, enrollment willingness, policy awareness, institutional trust, policy refinement

## Abstract

**Background:**

Achieving universal health coverage remains a global health priority. Understanding factors that influence individuals' willingness to enroll in basic medical insurance is essential for system sustainability.

**Objectives:**

This study aims to examine the impact of policy awareness and institutional trust on the willingness of urban and rural residents to enroll in the Urban and Rural Residents Basic Medical Insurance (URRBMI), with implications for policy refinement. The goal is to improve the precision of policy implementation and encourage broader insurance enrollment, supporting system sustainability.

**Methods:**

A survey of 563 residents was conducted in a county of Yunnan Province, China. Descriptive analysis of respondent demographics was followed by multiple linear regression, using policy awareness, policy expectation and institutional trust as independent variables, and demographics as control variables, to identify significant factors affecting enrollment willingness.

**Results:**

The results of the survey showed that 87.4% of respondents were enrolled in the URRBMI. The regression model, with an adjusted *R*^2^ of 0.322, showed policy awareness (coefficient = 0.243, *P* < 0.001) and institutional trust (coefficient = 0.354, *P* < 0.001) significantly and positively influenced enrollment willingness. Age and annual household income were also significant factors.

**Conclusion:**

Policy awareness and institutional trust are key determinants of enrollment willingness, with age and income playing roles. Policy education and communication should be refined, leveraging big data for targeted outreach and flexible payment options to enhance enrollment and system sustainability.

## Introduction

Social medical insurance is an essential element of social security, significantly contributing to the achievement of the World Health Organization member states' objective of universal health coverage (1), mitigating the economic burden on individuals and families during illness, preserving social stability, and advancing social equity. In China, the most extensively subscribed social medical insurance is the Urban and Rural Residents Basic Medical Insurance (URRBMI), which in 2023 covered a remarkable 962.94 million people (2).

The precursor to the URRBMI was a triad of concurrent social medical insurance systems: the Urban Employee Basic Medical Insurance (UEBMI), the New Rural Cooperative Medical System (NRCMS), and the Urban Residents' Basic Medical Insurance (URBMI) (3, 4). These three systems jointly achieved comprehensive coverage for both urban and rural residents, as well as urban employees, playing an essential role in aiding individuals to mitigate the risk of illness.

However, inherent flaws within such a system design became increasingly apparent over time. For instance, the NRCMS and the URBMI exhibited a distinct “urban-rural divide” characteristic (5), with notable disparities in their insured populations, funding mechanisms, and reimbursement benefits (6). Furthermore, the allocation of medical resources between urban and rural areas was markedly unbalanced (7), and the “fragmented” nature of the system compounded the complexities of medical insurance administration. Therefore, structural adjustments and integrations of the system emerged as a strategic and necessary choice.

In January 2016, the State Council of the People's Republic of China promulgated the “Opinions on Integrating the Basic Medical Insurance Systems for Urban and Rural residents” (8–10), embarking on the consolidation of the URBMI with the NRCMS into a unified URRBMI. The integration process successfully culminated in the national unification of the system by the end of 2019. Under this system, urban and rural residents, irrespective of their occupation, location, or income status, are afforded relatively equitable medical insurance services, signifying a considerable enhancement in both the fairness of medical resource allocation and the efficiency of utilization. For instance, the URRBMI has significantly increased the probability and extent of outpatient treatment benefits (11), while also notably improving the reimbursement treatment for the migrant workers (12). Moreover, research has been conducted on the satisfaction with the URRBMI (13, 14), as well as the perceived effect of financial risk protection by the insurance system (3).

**Figure 1:**
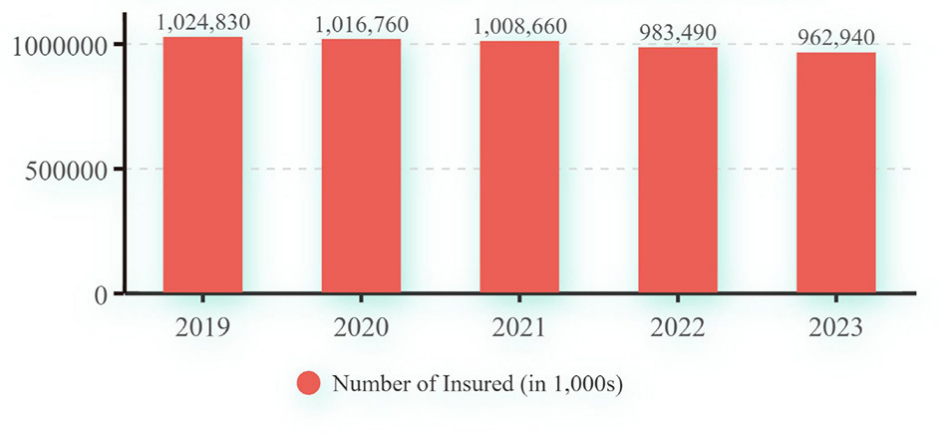
Number of enrollees in China's URRBMI from 2019 to 2023 (2).

However, in the midst of a research field burgeoning with diverse and flourishing contributions, a stark reality has come into focus: in recent years, the number of individuals enrolled in the URRBMI has exhibited a concerning trend of annual decline, as depicted in Figure 1.

The incremental decline in the number of insured individuals arguably reflects a diminishing inclination to subscribe, subsequently precipitating a decline in insurance enrollment behaviors. This downturn in enrollment could potentially lead to profound and pessimistic repercussions for the sustainability of the URRBMI. Hence, the significance of researching the willingness to engage with medical insurance, along with the factors that influence it, becomes increasingly evident. Specifically, enrollment in the URRBMI in China is contingent upon paying the stipulated annual premium. Thus, willingness to enroll in URRBMI inherently implies a willingness to pay for the scheme. Additionally, numerous studies have attempted to elucidate this particular dimension.

In a questionnaire-based study, Singu Hari Babu examined the willingness to pay (WTP) for customized health insurance package among 1,254 low-income households in India, alongside the factors influencing this willingness. Regression outcomes demonstrated an increased propensity to pay as educational levels rose, and a corresponding rise in payment readiness in households with a greater count of dependents and sick family members (15).

In northern Ethiopia, a study among 823 families found a strong willingness to pay for community-based health insurance. The researchers identified perceived affordability and households' awareness of their own monthly income—referring to their ability to accurately assess and understand their income levels—as pivotal factors influencing the intention to enroll (16).

In Cameroon, a study found that factors such as higher household income, larger family size, prioritizing health, and formal employment were associated with a greater willingness to join both compulsory (CCHI) and voluntary (VCHI) community-based health insurance schemes. Notably, for voluntary schemes, households with more children were willing to pay higher premiums, whereas the presence of chronic illness reduced willingness to pay for VCHI (17).

A survey conducted in Sierra Leone identified factors influencing WTP for health insurance. The findings highlighted five key predictors: household monthly income, age, district of resident, gender, and educational qualification. Specificlly, higher income and secondary school education significantly increased WTP, while older individuals (60+ years) and male respondents were less likely to pay. Residents in Bo district exhibited higher WTP, whereas those in Koinadugu district showed reduced willingness (18).

Additionally, existing studies have shown that WTP for social health insurance (SHI) is influenced by various psychosocial factors. Abate et al. conducted a study in Ethiopia, revealing that although 93.9% of bank employees had knowledge of SHI policies, only 50.4% expressed willingness to pay. The primary barriers included limited benefit coverage, shortages of medicines, and low-quality healthcare services. WTP was significantly influenced by gender [lower among females, adjusted odds ratio (AOR) = 0.50], religious affiliation (lower among Orthodox Christians, AOR = 0.48), family size (lower in households with ≥5 members, AOR = 0.17), recent illness experience (higher among those with illness in the past 6 months, AOR = 4.95), and perceptions of the policy (AOR = 4.07) (19).

These studies encompass multiple factors affecting individuals' willingness to pay for health insurance across varied income levels and socio-economic backgrounds. Nonetheless, research gaps remain concerning specific issues related to the willingness to enroll in medical insurance. For instance, in China, how policy awareness and institutional trust influence the willingness to enroll in the URRBMI and the policy implications that arise from this are not fully understood. This paper endeavors to bridge such gaps by examining how these two factors affect the insurance enrollment intentions of urban and rural residents. Through this research, valuable insights can be offered to continuously refine the system, assisting administrative agencies in enhancing the precision of policy implementation. Consequently, this could lead to increased enrollment in the URRBMI and provide robust support for the system's sustainable development.

## Methods

### Study design and setting

This study adopted a cross-sectional survey design and was conducted in a county located in Yunnan Province, China. This county was selected due to its average economic level within the province and the research team's familiarity with local health insurance operations.

### Theoretical framework and operational definitions

This study is grounded in the Theory of Planned Behavior (TPB) developed by Ajzen and Fishbein (20, 21), which posits that behavioral intention is jointly determined by three components: attitudes toward the behavior, subjective norms, and perceived behavioral control (21–23). Attitude reflects an individual's evaluation of the behavior, subjective norm refers to perceived social pressure from important others, and perceived behavioral control indicates one's perception of their ability to perform the behavior.

In our context, the intention to enroll in URRBMI is conceptualized as willingness to enroll in the scheme and each TPB component's operational definition is as follows.

Attitude is reflected in institutional trust, representing respondents' positive or negative evaluation of the URRBMI system.

Subjective norm is captured by social influence, including peer, family, and community recommendations, reflecting the perceived social pressure to enroll or not enroll.

Perceived behavioral control reflects individuals' perceived ability to enroll in URRBMI, which can be regarded as policy awareness in this study.

The dependent variable was the “willingness to enroll” in URRBMI.

Self-reported health status was categorized as “Poor,” “Fair,” “Good,” or “Excellent,” with “Good/Excellent” merged as favorable.

Demographic variables included gender, age, marital status, education, occupation, income, registered residence, health condition, and insurance enrollment.

### Sampling method

A stratified cluster sampling approach was used. Between December 2022 and February 2023, three townships were randomly selected from a county, Yunnan Province, China. Within each selected township, two villages or communities were further randomly chosen as sampling units. Within units, all eligible household members were invited. In total, 580 invitations were disseminated via community investigators, with 568 questionnaires returned (response rate: 97.9%). After data quality checks, 563 valid responses were included.

### Questionnaire design and measurement

The structured questionnaire comprised five sections. The first section collected demographic information. These variables were used as controls in the analysis.

Sections 2 to 5 each contained three items measured on a 5-point Likert scale (1 = strongly disagree to 5 = strongly agree).

Section 2 assessed willingness to enroll in URRBMI (dependent variable), with items developed based on prior research on insurance enrollment behavior (13, 24).

Section 3 measured policy awareness, adapted from established studies on health policy awareness (4).

Section 4 evaluated policy expectations, based on national URRBMI policy frameworks and prior research (25).

Section 5 assessed institutional trust, with items adapted in consultation with relevant literature (13, 26).

Scale scores were calculated as the mean of the corresponding items. This design allowed for a comprehensive and reliable assessment of the main constructs under investigation.

### Data collection and quality control

Trained investigators distributed and collected the electronic questionnaires. A pilot test with 20 residents led to minor revisions for clarity. After data collection, responses were screened for completeness and consistency; those with major inconsistencies or over 20% missing data were excluded. Data were double-checked before analysis, yielding 563 valid questionnaires.

### Sample size calculation

The sample size was determined based on the following formula:


$$\begin{array}{l} n=\frac{z^{2}\;*\;p\;*\;\left(1-p\;\right)}{d^{2}} \end{array}$$

For sample size estimation, a 95% confidence level (z = 1.96) was used. The proportion (p) was set at 0.5 due to a lack of prior data, with a margin of error (d) of 0.05. This yielded a minimum sample of 385 respondents. To ensure that the final number of valid responses met the calculated minimum requirement of 385, and to account for anticipated factors such as non-response, incomplete surveys, or invalid entries which are common in survey research, 580 questionnaires were distributed. A total of 568 completed questionnaires were received. After cleaning, 563 valid questionnaires were included.

### Statistical analysis

Data were analyzed using SPSS version 26.0. Questionnaire reliability and validity were assessed, followed by descriptive analyses of demographic characteristics. Multivariate linear regression was conducted with policy awareness, policy expectation, and institutional trust as independent variables, controlling for demographics, to identify determinants of willingness to enroll in the URRBMI. Model fit and coefficient significance were evaluated to determine key influencing factors.

### Reliability and validity assessment

Reliability reflects the consistency and stability of a measurement tool (27, 28) and is essential for assessing measurement quality. In this study, Cronbach's alpha was used to evaluate the reliability of the scales for willingness to enroll, policy awareness, policy expectation, institutional trust, and the overall questionnaire. A Cronbach's alpha above 0.9 indicates excellent reliability, 0.8–0.9 good reliability, and 0.7–0.8 acceptable reliability. However, a value above 0.9 is not always optimal for all scales, with some studies suggesting ≥0.6 as acceptable (29, 30). Table 1 displays the SPSS output for Cronbach's alpha.

**Table 1:** Reliability test.

**Dimension**	**Cronbach's alpha**	**Number of items**
Willingness of enrollment	0.846	3
Policy awareness	0.836	3
Policy expectation	0.862	3
Institutional trust	0.854	3
Overall questionnaire	0.889	12

Table 1 presents the reliability analysis (Cronbach's alpha coefficients) for each dimension of the questionnaire, which measured willingness of enrollment, policy awareness, policy expectation, and institutional trust. Data were collected from participants in a county in Yunnan Province, China, between December 2022 and February 2023. All dimensions yielded Cronbach's alpha coefficients above 0.8, indicating high reliability and stability of the questionnaire.

Validity indicates how well a measurement tool captures the intended construct (31), which is critical for research credibility (32). The questionnaire's suitability for factor analysis and discriminant validity was assessed using the Kaiser–Meyer–Olkin (KMO) measure and Bartlett's test of sphericity, based on data from 563 residents in a county of Yunnan Province, China (December 2022–February 2023). The KMO value was 0.880 (>0.8), and Bartlett's test was significant (*P* < 0.001) shown in Table 2, supporting the questionnaire's validity for factor analysis.

**Table 2:** KMO and Bartlett's test.

**KMO measure of sampling adequacy**		**0.880**
Bartlett's test of sphericity	Approx. chi-square	3,527.116
	Df	66
	Sig.	0.000

## Results

### Demographic characteristics of participants

This study collected data through a survey conducted in a specific county within Yunnan Province, China, between December 2022 and February 2023. The following demographic characteristics describe the participants (*n* = 563) who completed the survey, as detailed in Table 3.

**Table 3:** Demographic characteristics of participants (*n* = 563).

**Variables**	***N***	**%**
**Gender**		
Male	276	49
Female	287	51
**Age**		
<18	5	0.9
18–25	99	17.6
26–35	135	24
36–45	159	28.2
46–55	116	20.6
56–65	32	5.7
>65	17	3
**Marital status**		
Unmarried	204	36.2
Married	359	63.8
**Education**		
Primary school	52	9.2
Middle school	71	12.6
High school/secondary vocational school	72	12.8
Associate degree	135	24
Bachelor's degree	201	35.7
Master's degree and above	32	5.7
**Occupation**		
Self-employed	53	9.4
Student	63	11.2
Service industry	32	5.7
Company employee	256	45.5
Government employee	86	15.3
Farmer	36	6.4
Other	37	6.6
**Annual household income level**		
<50,000 RMB	145	25.8
50,000–100,000 RMB	185	32.9
100,001–150,000 RMB	132	23.4
150,001–200,000 RMB	64	11.4
>200,000 RMB	37	6.6
**Registered residence**		
Urban registered residence	312	55.4
Rural registered residence	251	44.6
**Health condition**		
Poor	18	3.2
Fair	140	24.9
Good	228	40.5
Excellent	177	31.4
**Insurance enrollment**		
Yes	492	87.4
No	71	12.6

The gender distribution among the subjects was relatively even, with 276 males (49%) and 287 females (51%). The ages of the participants were mainly between 26 and 55 years, with the largest subgroup being those between 36 and 45 years old, accounting for 159 individuals or 28.2% of the total respondents. In terms of marital status, over 60% were married. Regarding education, the majority had received associate or bachelor's degrees, totaling 336 individuals (59.7%), while those with middle school education or below made up 21.8%. Occupational distribution indicated that company employees formed the largest group (45.5%). Households with annual incomes of 50,000–100,000 RMB represented 32.9%. Of the surveyed subjects, 55.4% had urban registration and 44.6% had rural registration. The health conditions for most were favorable, with “Good” and “Excellent” together accounting for 71.9%, and a notable 87.4% of participants were enrolled in the URRBMI.

### Evaluation of regression model assumptions

We assessed the key assumptions of multiple linear regression: homoscedasticity, linearity, normality and randomization. Residual scatter plots showed the residuals were evenly distributed without discernable patterns, indicating homoscedasticity and linearity. The distribution of standardized residuals, as assessed by both a histogram and a P-P plot, was approximately normal. Random sampling was used for data collection, and analysis of variance (ANOVA) confirmed no significant differences between sample groups, supporting the randomization assumption. Overall, all core assumptions were met, demonstrating the robustness and suitability of the regression model.

### Results of multiple linear regression

To identify the factors significantly associated with willingness to enroll in the URRBMI among residents in this county of Yunnan Province, China, a multiple linear regression analysis was conducted using the data collected between December 2022 and February 2023. The model included policy awareness, policy expectation, institutional trust, and various demographic characteristics as independent variables. The dependent variable was willingness to enroll in the URRBMI, which was measured quantitatively using a five-point Likert scale, where one represented “very unwilling” and five “very willing.” The resulting scores were treated as a continuous variable for the regression analysis. Table 4 presents the results of this regression analysis.

**Table 4:** Multiple linear regression on influencing factors of willingness to enroll in the URRBMI.

**Variables**	**Unstandardized coefficient *B***	**Standardized coefficient beta**	***t***	***P*-value**	***F***	**Adjusted *R*^2^**
**Constant**	1.566		5.188	0.000	9.615 (*P* < 0.001)	0.322
Policy awareness	0.243	0.265	6.124	0.000		
Policy expectation	0.019	0.022	0.486	0.627		
Institutional trust	0.354	0.385	7.350	0.000		
**Gender**				
Male	0.048	0.029	0.814	0.416		
Female (reference)	0					
**Age**				
<18	0.112	0.013	0.289	0.773		
18–25	0.333	0.155	1.513	0.131		
26–35	0.516	0.270	2.843	0.005		
36–45	0.357	0.197	1.927	0.054		
46–55	0.616	0.306	3.378	0.001		
56–65	0.809	0.230	3.897	0.000		
>65 (reference)	0					
**Marital status**				
Unmarried	0.148	0.087	1.527	0.127		
Married (reference)	0					
**Education**				
Primary school	−0.024	−0.008	−0.145	0.884		
Middle school	−0.123	−0.050	−0.797	0.426		
High school/secondary vocational school	−0.102	−0.042	−0.677	0.499		
Associate degree	−0.051	−0.027	−0.363	0.717		
Bachelor's degree	−0.022	−0.013	−0.162	0.871		
Master's degree and above (reference)	0					
**Occupation**				
Self-employed	−0.053	−0.019	−0.322	0.748		
Student	−0.014	−0.005	−0.084	0.933		
Service industry	−0.266	−0.075	−1.507	0.132		
Company employee	−0.059	−0.036	−0.430	0.667		
Government employee	−0.037	−0.016	−0.241	0.810		
Farmer	−0.225	−0.068	−1.267	0.206		
Other (reference)	0					
**Annual household income level**				
<50,000 RMB	−0.219	−0.117	−1.623	0.105		
50,000–100,000 RMB	−0.367	−0.212	−2.817	0.005		
100,001–150,000 RMB	−0.281	−0.146	−2.086	0.037		
150,001–200,000 RMB	−0.242	−0.094	−1.657	0.098		
>200,000 RMB (reference)	0					
**Health condition**				
Poor	0.068	0.015	0.387	0.699		
Fair	−0.030	−0.016	−0.364	0.716		
Good	0.005	0.003	0.078	0.938		
Excellent (reference)	0					
**Registered residence**				
Urban registered residence	0.012	0.007	0.170	0.865		
Rural registered residence (reference)	0					
**Insurance enrollment**				
Yes	0.017	0.007	0.175	0.861		
No (reference)	0					

Dependent variable: enrollment willingness.

The SPSS output indicated a good model fit, with an adjusted *R*^2^ of 0.322, which suggested that the independent variables included in the regression analysis accounted for 32.2% of the variation observed in the dependent variable. In other words, 32.2% of the changes in urban residents' willingness to enroll in the URRBMI can be explained by the predictors in the current regression model. Overall, the model provided a reasonably good examination of the factors influencing residents' enrollment willingness.

The linear regression model in this study is significant with *F* = 9.615, *P* < 0.001, indicating that at least one of the predictors significantly influences the outcome variable of enrollment willingness. Furthermore, combined with tests on the regression coefficients of the predictors, it can be concluded that.

Policy awareness significantly and positively influenced enrollment willingness, with a coefficient of 0.243 (*t* = 6.124, *P* < 0.001). This indicates that higher levels of policy awareness are associated with stronger willingness among urban and rural residents to enroll in basic medical insurance. Quantitatively, one unit increase in policy awareness is accompanied by 0.243 unit increase in enrollment willingness.

Institutional trust was found to exert a significant positive influence on enrollment willingness, as indicated by a coefficient of 0.354 (*t* = 7.35, *P* < 0.001). Higher institutional trust correlates with increased willingness among urban and rural residents to enroll in basic medical insurance. Quantitatively speaking, enrollment willingness rises by 0.354 units with each single unit increment in institutional trust.

Additionally, age group as a control variable had a significant influence on enrollment willingness. Specifically, people aged 26–35, 46–55, and 56–65 years exhibited markedly higher willingness than those over 65 years old. Among these groups, individuals aged 56–65 years showed a 0.809 unit (*t* = 3.897, *P* < 0.001) greater level of enrollment willingness on average compared to the over 65 group.

Annual household income also exhibited a significant influence on enrollment willingness. Specifically, people with ¥50,000–100,000 and ¥100,001–150,000 annual income showed markedly lower willingness than those above ¥200,000. Among these groups, individuals with ¥50,000–100,000 income had a 0.367 unit lower level of enrollment willingness on average compared to the above ¥200,000 group (*t* = −2.817, *P* = 0.005 <0.05). Meanwhile, people with ¥100,001–150,000 income averaged 0.281 units lower in willingness vs. the above ¥200,000 group (*t* = −2.086, *P* = 0.037 <0.05).

All relevant categorical and nominal variables were included in the multiple linear regression model using dummy coding, with one category of each variable serving as the reference group. The regression analysis identified the predictors that significantly influenced enrollment willingness at the 0.05 significance level (Table 4).

## Discussion

In this survey, 87.4% of participants were enrolled in the URRBMI. Although this proportion didn't reflect the actual enrollment rate across the county, it indicated a relatively high willingness among local residents to enroll. This could be attributed to the respondents' high awareness of insurance policies and trust in the system, as evidenced by the multiple linear regression analysis. Both policy awareness and institutional trust were found to exert significant positive influences on enrollment willingness (19, 33). This signifies the importance of maintaining or even strengthening efforts in policy promotion and education, as well as ensuring fairness in policy implementation (10) and transparency of outcomes (6), in order to improve residents' willingness to enroll.

As mentioned earlier, TPB posits that behavioral intention is determined by three key components: attitudes toward the behavior, subjective norms, and perceived behavioral control (21, 34). This study has validated the applicability of this theory.

More precisely, both policy awareness (perceived behavioral control) and institutional trust (attitudes) were found to have a significant positive influence on the willingness of enrollment in the URRBMI (behavioral intention), which is a key factor in developing favorable attitudes (23, 35). Residents who possess a greater understanding of policy context, premium rates and duration, benefits coverage, and the relief of disease economic burden, are better equipped to develop an informed and positive understanding of the importance and necessity of the system, thereby reinforcing their willingness to enroll. The importance of institutional trust cannot be understated either. The stronger the residents' sense of trust toward the medical insurance administration and staff, the more their willingness to enroll will intensify accordingly. This trust may stem from people's comprehensible perceptions and experiences in practice, such as the smoothness of information channels and accessibility of information, the effectiveness of public participation in policy making and improvement, and civil supervision of policy implementation. Moreover, policy awareness and institutional trust demonstrated a positive correlation in this study, with a Pearson correlation coefficient of 0.503 (*P* < 0.01). This implies that policy awareness and institutional trust promote each other to some extent, both serving as potent motivational factors in attitude formation, and thereby exerting significant influences on rural and urban residents' willingness to enroll in basic medical insurance.

In addition, age group and annual household income were found to have significant influences on enrollment willingness. Individuals aged 26–35, 46–55, and 56–65 years demonstrated higher willingness compared to those above 65 years old. This could be attributed to the fact that the majority of individuals in the former three age groups are still in the stages of career development and raising a family. The pressures and needs during these stages may positively reinforce their willingness to enroll. On the other hand, individuals above 65 years old experience an increase in demand for medical insurance as they grow older. However, since most individuals retire from work at this age, declining income becomes a limiting factor, resulting in weaker enrollment willingness compared to the other three age groups. A similar finding was reported by Agyei-Baffour et al. (18) who identified age as a predictive factor for people's willingness to pay health insurance premiums in Sierra Leone, and older individuals (60+ years) and male respondents were less likely to pay.

On the other hand, people with annual household income exceeding 200,000 RMB exhibited a significantly higher willingness of enrollment compared to those with annual incomes ranging from 50,000–100,000 to 100,001–150,000 RMB. This may be attributed to the fact that as income increases, so does the individual's awareness of medical security, leading to a stronger inclination to enroll in insurance and the financial capacity to do so. Similar findings have been reported in other studies (16–18). This observation may suggest that policies should be refined to provide additional support for disadvantaged groups, such as the older adult and low-income population, ensuring their access to essential medical security and uphold the fairness of the system.

The theoretical framework of TPB suggests that subjective norms also influence behavioral intention (21, 36). In the survey, a question was designed: “I have great trust in community (village) officials. If they recommend that I enroll in the URRBMI, I would be willing to try.” This question was used to examine the relationship between subjective norms and enrollment willingness (behavior intention). When analyzing the correlation between this question and enrollment willingness, we found that the Pearson correlation coefficient was 0.426 (*P* < 0.01), indicating a moderate positive correlation. This result suggests that, under the premise of trust in community (village) officials, their recommendations significantly contribute to shaping individuals' willingness to enroll in the insurance program, aligning with the TPB framework's assumption that subjective norms, derived from social pressure or external influences, play an important role in influencing behavioral intention (37).

Additionally, the survey process revealed a significant phenomenon that cannot be overlooked: many individuals have been making annual contributions, with the personal payment standards increasing annually. Taking Yunnan Province as an example, the individual premium rate rose from 250 RMB in 2020 to 400 RMB in 2025. However, many of these insured members have not been ill or claimed reimbursement over the past 5 years or longer, resulting in only unilateral premium payment without receiving benefits in return. Survey respondents may perceive it as pure expense without corresponding compensation, which could undermine their willingness to continue enrolling in the insurance scheme. During the survey, many respondents expressed similar thoughts. The annually rising individual premium rates could be seen as negative external incentive. Without corresponding health reimbursement benefits for a considerable period, individual intrinsic motivation is undoubtedly negatively affected, posing a potential threat to the sustainable development of the insurance system. Studies have also indicated that individuals' WTP decreases as the price of health insurance increases (38–40). Therefore, along the path of reshaping the core values of medical insurance to be markedly different from a simplistic exchange-for-benefits view, it is imperative and pressing to tap the potential and pertinence of education and publicity more effectively.

To address these issues, the government could take some measures. First, education and promotion methods should be optimized, for example by adopting a segmentation strategy (41–43). For information dissemination channels and media choice, young people have greater exposure to computers and mobile phones, hence policy details and interpretations about the URRBMI could be publicized on mainstream portal websites and frequently-used mobile apps. Considering older adult's media exposure habits, in addition to broadcasting related policies on television and radio, lectures could be conveniently organized in local communities to provide direct access to policy awareness for seniors.

Secondly, the content of the health insurance policy can be presented in more engaging and interactive formats such as animation and video (44, 45). These formats can effectively showcase the core values of social health insurance, contributions, reimbursement details, and benefits through storytelling and examples. This approach can help deepen the understanding of the health insurance policy among urban and rural residents and strengthen their willingness to enroll in the scheme. Additionally, it is important for relevant departments to utilize the statistical functions of mainstream portals and apps to analyze the online behavior and habits of current and potential participants (46, 47). By leveraging big data mining and matching algorithms (48–50), it is possible to analyze the number of clicks on articles, videos, and animations related to health insurance policies, as well as the level of interactive forwarding and sharing. This data-driven approach could enable accurate targeting of the intended audience.

Finally, during the ongoing optimization of medical insurance policies, it may be beneficial to explore the introduction of flexible methods (51). For instance, a points-based system could be implemented for the insured population, where certain incentives are provided based on their payment and reimbursement history. If an individual consistently contributes without making any claims for several consecutive years, a gradual reduction in their contribution rate could be applied in subsequent insurance years. This approach aims to mitigate the negative external incentives and enhance their motivation and willingness to continue enrolling in the URRBMI.

## Limitations of the study

Despite attempts in this study to explore the willingness to enroll in the URRBMI from the perspectives of policy awareness and institutional trust, certain limitations were inevitable. Firstly, there was a constraint regarding the source of the sample. All respondents were from a single county in Yunnan Province, which has a moderate level of economic development, hence the representativeness of the findings may be restricted and potentially not generalizable to other regions. Secondly, the data from survey questionnaires might be subject to some subjective bias. During the survey, respondents' comprehension of policy details and the implementation process might be limited due to factors such as their educational background, personal experiences, or other influences, leading to a lack of objective consistency in their responses, which could introduce a degree of bias in the questionnaire data. Lastly, other factors that might influence the willingness of urban and rural residents to enroll, such as the quality of medical services, the strength of social support networks, and variations in social expectations among residents, were not considered in this study. These could be significant determinants of enrollment intentions but were not accounted for. Future research should aim to expand the geographic scope of the sample survey, incorporate more variables and employ mixed-methods approaches (combining questionnaires with qualitative in-depth interviews) to improve the practical applicability of the study and provide robust support for the optimization of health insurance policies.

## Conclusion

This study identified several factors significantly associated with the willingness enroll in the URRBMI. Policy awareness and institutional trust were found to have a significant positive relationship with willingness to enroll. Age and annual household income also showed significant associations. Additionally, the willingness to remain insured may decrease among those who have paid premiums for many years without submitting claims. These findings suggest that relevant authorities could improve educational and promotional strategies, enhance information dissemination, and leverage big data analytics to better target and expand coverage. Furthermore, implementing flexible contribution measures may encourage continued enrollment willingness and thus support the sustainable development of the system.

## Data Availability

The raw data supporting the conclusions of this article will be made available by the authors, without undue reservation.
