# Supplementary material for: Study on the willingness to enroll in the urban and rural residents basic medical insurance: from the perspectives of policy awareness and institutional trust
Source: Front Public Health. 2025 Jun 24;13:1535558. doi: 10.3389/fpubh.2025.1535558 (PMC12234447; doi:10.3389/fpubh.2025.1535558)
Supplement: Supplementary file 1 [file Data_Sheet_1.pdf]

Survey on Factors Influencing the Willingness to Participate in the Urban and Rural Residents Basic Medical Insurance (URRBMI)

Dear Participant:

Hello! To better understand the willingness of urban and rural residents to enroll in the Urban and Rural Residents Basic Medical Insurance (URRBMI) program and the factors that influence it, as well as to improve the relevance of the insurance policies and the quality of services, we are conducting this survey. There are no right or wrong answers in this questionnaire, so please feel free to answer based on your actual situation. All of your information will be kept strictly confidential and will be used solely for statistical analysis. Thank you for taking the time to participate in this survey!

Demographic Information

1.What is your gender?

A. Male

B. Female

2.What is your age?

A. Under 18

B. 18–25

C. 26–35

D. 36–45

E. 46–55

F. 56–65

G. Over 65

3.What is your marital status?

A. Unmarried

B. Married

4.What is your highest level of education?

A. Primary school

B. Middle school

C. High school / Secondary vocational school

D. Associate degree

E. Bachelor’s degree

F. Master’s degree and above

5.What is your current occupation?

A. Self-employed

B. Student

C. Service industry

D. Company employee

E. Government employee

F. Farmer

G. Other

6.What is your annual household income level?

A. Less than 50,000 RMB

B. 50,001–100,000 RMB

C. 100,001–150,000 RMB

D. 150,001–200,000 RMB

E. Over 200,000 RMB

7.What is your registered residence?

A. Urban registered residence

B. Rural registered residence

8.What is your current health condition?
A. Poor
B. Fair
C. Good
D. Excellent

9.Have you enrolled in the URRBMI program before?
A. Yes
B. No

10.What are your primary sources of information about the URRBMI program? (Multiple choice)
A. Official brochures or medical guides
B. Official websites or official WeChat accounts of the Human Resources and Social Security Bureau, Medical Insurance Bureau, or Tax Bureau
C. Relevant medical institutions
D. News media
E. Other online platforms
F. Classmates or friends
G. Others

Evaluation of Participation Willingness and Policy Cognition of URRBMI

Dimension	Questions	Strongly Disagree	Disagree	Neutral	Agree	Strongly Agree
Participation Willingness	11. I am willing to enroll in the URRBMI.	1	2	3	4	5
	12. I would recommend	1	2	3	4	5

	my neighbors, relatives, and friends to enroll in the URRBMI.					
	13. I will continue to participate in the URRBMI.	1	2	3	4	5
Dimension	Questions	No Awareness at All	Limited Awareness	Not Sure	Awareness	Comprehensive Awareness
Policy Awareness	14.Are you aware of the validity period of the URRBMI?	1	2	3	4	5
	15. Are you aware of the individual contribution standards for the URRBMI?	1	2	3	4	5
	16. Are you aware of the benefits brought by the URRBMI?	1	2	3	4	5

Dimension	Questions	Strongly Disagree	Disagree	Neutral	Agree	Strongly Agree
Policy expectation	17. The government will strengthen the promotion of the URRBMI.	1	2	3	4	5
	18. The range of reimbursable medicines under the URRBMI will expand.	1	2	3	4	5
	19. More and more people will participate in the URRBMI.	1	2	3	4	5
Dimension	Questions	Strongly Disagree	Disagree	Neutral	Agree	Strongly Agree
Institutional trust	20.I have great trust in community (village) officials. If they recommend that I	1	2	3	4	5

	participate in the URRBMI, I would be willing to try.					
	21. I have great trust in the policies and systems of the URRBMI, and I believe the government provides strong supportive systems and policies.	1	2	3	4	5
	22. I have great trust in the relevant policies and regulations of the URRBMI, and I believe everyone will abide by these policies and systems.	1	2	3	4	5
